# 96 How Many Lives Did Your Program Save? Defending Healthcare Epidemiology, Protecting Patients

**DOI:** 10.1017/ash.2026.10518

**Published:** 2026-06-23

**Authors:** Dan Ilges, M. Teresa Seville, Jenna Reynolds, Sandhya Nagarakanti, Jamilah Shubeilat, Valerie Armstrong, Pete Johnston, Drew Dickinson

**Affiliations:** 1 Mayo Clinic Arizona; 2 Mayo Clinic; 3 The Mayo Clinic Hospital - Arizona

## Abstract

**Background:** Handshake antimicrobial stewardship (AMS), characterized by regular, face-to-face interactions with antimicrobial prescribers with broad review of all active antimicrobials, is considered an AMS best practice. This approach fosters relationship building and increases opportunities for targeted education compared to traditional AMS activities. Reports of handshake AMS within surgical services are limited. We evaluated the impact of once-weekly handshake AMS rounds among general surgery teams in a 368-bed academic medical center. **Methods:** An infectious diseases physician and pharmacist conducted weekly rounds in general surgery offices with surgical residents, chief residents, advanced practice providers (APPs), medical students, and occasionally attending surgeons. All patients on antimicrobials and without an active ID consult were reviewed and recommendations with targeted education were provided. The number, type, and acceptance rate of recommendations were collected prospectively. Antimicrobial utilization within general surgery was reviewed pre- (1/1/2024-12/31/2024) and post-implementation (1/1/2025-12/31/2025) of handshake AMS rounds. Antimicrobial use data are presented as days of therapy (DOT)/1000 days present (DP). **Results:** From January 7, 2025, through January 6, 2026, 47 handshake AMS sessions were performed. Over this period, 215 recommendations were provided, and 173 recommendations were implemented, yielding a recommendation implementation rate of 80.5% and an average of 4.6 recommendations/session. The most common accepted recommendation types were duration modification (n=42, 24.3%), antibiotic stop (n=38, 22.0%), and de-escalation (n=33, 19.1%). Average DOT/1000 DP on general surgery teams for all agents dropped from 481.3 pre- to 462.8 post-implementation (3.8% relative reduction, p=0.578). Broad-spectrum antibiotic use dropped by 17.5%, from 369.2 pre- to 304.6 post-implementation (p=0.055). Anti-MRSA agent utilization remained stably low, with an average of 45.4 pre- vs 49.0 DOT/1000 DP post-implementation. The use of enteral metronidazole increased, from 4.9% pre- to 18.2% enteral/IV DOT post-implementation. Surgical providers consistently expressed appreciation for the practice-changing education provided during handshake AMS rounds and the resulting enhancements in antimicrobial decision-making and patient care. **Conclusion:** Targeted, once-weekly handshake AMS rounds with general surgery resulted in high-yield interventions with a high acceptance rate. Handshake AMS rounds could be an effective stewardship intervention in surgical practices and is doable despite the challenges of unpredictable operating room/provider schedules.

